# Diabetes Insipidus as a Complication of Wegener's Granulomatosis and Its Treatment with Biologic Agents

**DOI:** 10.1155/2009/346136

**Published:** 2009-07-26

**Authors:** Joanna Rosalind Cunnington, Ramesh Jois, Ivan Zammit, David Scott, John Isaacs

**Affiliations:** Musculoskeletal Research Group, Institute of Cellular Medicine, The Medical School, Framlington Place, Newcastle upon Tyne, NE2 4HH, UK

## Abstract

Wegener's granulomatosis of the pituitary gland resulting in diabetes insipidus is a rare complication of the disease. Standard treatment for Wegener's granulomatosis involves a combination of prednisolone and cylophosphamide, however biologic agents are now being used in refractory cases. We report three cases of patients with diabetes insipidus as a complication of Wegener's granulomatosis who were treated with biologic agents. All three cases showed clinical response to treatment with biologic agents including rituximab and alemtuzumab and two cases demonstrated improvement in pituitary gland abnormalities by MRI. Clinicians should be aware that diabetes insipidus can present as a complication of Wegener's granulomatosis and that biologic therapies may be effective in refractory cases.

## 1. Introduction

Wegener's granulomatosis (WG) is a systemic, necrotizing, granulomatous vasculitis of unknown aetiology. Diffuse small and medium vessel involvement in WG can result in a wide range of clinical manifestations, however classically WG affects the upper and lower respiratory tracts and kidneys. Central nervous system (CNS) involvement in WG is unusual, and diabetes insipidus (DI) secondary to pituitary involvement in WG is rare [1]. Standard therapy for aggressive WG remains a combination of prednisolone and cyclophosphamide, a regime that has been shown to induce remission and reduce mortality [2]. However not all patients respond to this regime so alternative therapeutic options are being investigated. In this report we present three cases of DI secondary to WG that have required biologic therapies to achieve remission.

## 2. Case 1

A 19-year old man presented in July 2000 with recurrent epistaxis, haemoptyis, nasal crusting, vasculitic skin rash, and bilateral episcleritis. On admission he was systemically unwell with a high ESR (113 mm/h) and CRP (61 mg/L). Full blood count and renal function were normal. Chest x-ray showed bilateral pulmonary cavities. Urinalysis was positive for blood and protein. Cytoplasmic antineutrophil cytoplasmic antibody (cANCA) was positive with a PR3 titre of 73 (normal <6). Skin biopsy was in keeping with WG. Mycobacterial and fungal infection were excluded. 

 He was started on intravenous pulses of cyclophosphamide alongside prednisolone (initially 40 mg per day) and co-trimoxazole. His disease rapidly responded. ESR fell to 15 mm/h, CRP to 22 mg/dL, and PR3 cANCA titre to 15. Urinalysis became negative, and chest x-ray showed complete resolution of pulmonary cavities. He continued to suffer low-grade nasal and sinus symptoms and, over the next four years, received methotrexate (up to 25 mg/week), infliximab (up to 5 mg/kg 6 weekly), and mycophenylate mofetil (1 g twice daily), interspersed with further courses of pulsed or oral cyclophosphamide and prednisolone. Despite these measures, he continued to have clinically active ENT disease, low-grade inflammatory markers, and MRI evidence of gradually progressive destruction of sinuses and nasal septum. 

 In May 2005 he developed polyuria and polydypsia. Investigations showed serum osmolarity of 303 mosmol/kg (normal up to 295) and urine osmolality of 75 mosmol/kg (normal 40–1200), serum creatinine 92 *μ*moL and sodium 142 mmol/L (urinary sodium not available). MRI scan showed inflammation involving the sphenoid sinus and left cavernous sinus, dural enhancement and infiltration, and enlargement of the pituitary gland. Circulating levels of anterior pituitary hormones were within normal limits. The diagnosis of diabetes insipidus secondary to pituitary infiltration from WG was made. A water deprivation test was not performed because of the striking clinical presentation and the dramatic response to desmopressin (polyuria/polydypsia resolved, serum and urine osmolarity returned to normal). 

 In view of the progression of the underlying WG despite conventional therapies, he was treated with rituximab 1 g intravenously on two occasions 15 days apart. Mycophenolate mofetil was discontinued four weeks after the second dose. He received a second cycle of rituximab 15 months after the first one for presumed neurological recurrence-memory loss, sleep disturbance, and seizures, all of which later improved. Repeat MRI scan done 17 months later showed normalization of the previously described pituitary changes. In addition there was no further progression of the disease in the sinuses or intracranially. The dural enhancement that was seen earlier was also not seen now. A third MRI scan done another 15 months on continues to be stable with normal appearance of the pituitary. In this time there have been no flares of his systemic WG. The most recent investigations show an ESR 11 mm/h, CRP 3 mg/dL and PR3 ANCA titre of 12. Prednisolone dose has been reduced to 5 mg daily. He however continues to use desmopressin and sodium valproate.

## 3. Case 2

A 33-year old female presented in 2002 with a 10 week illness comprising bilateral otalgia, otorrhoea, hearing loss, epistaxis, anorexia, and weight loss. On admission ESR was 84 mm/h, CRP 224 mg/L, and cANCA titre 1 in 160 (MPO and PR3 not available). Haemoglobin and renal function were normal. Urine dipstick was positive for blood and protein. Chest x-ray was normal. A nasal biopsy revealed extensive tissue necrosis, florid, active chronic inflammation and widespread, and severe transmural active vasculitis consistent with WG. She was treated for seven months with oral prednisolone, oral cyclophosphamide (150 mg daily), and co-trimoxazole. Following clinical improvement, she was converted to azathioprine and prednisolone maintenance therapy. Her symptoms remained under control, and cANCA titre remained either negative or very low. 

**Figure 1:**
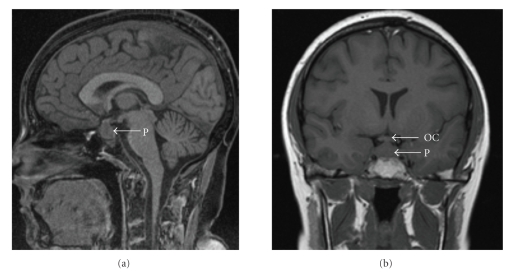
(a) Sagital volumetric interpolated breath hold examination (VIBE) image demonstrating enlarged pituitary with loss of T1W increased signal in the posterior pituitary lobe consistent with diabetes insipidus. (b) Coronal T1W image demonstrating the enlarged pituitary in relation to the optic chiasm. OC : optic chiasm and P : pituitary.

 In 2003 she presented with polyuria, polydypsia, and severe frontal headache. There were no neurological signs on examination. Investigations showed ESR 26 mm/h, CRP 10 mg/L, cANCA 1 in 80, serum sodium 141 mmol/L, serum creatinine 87 *μ*mol/L, serum osmolality 300 mosmol/kg, urinary sodium 58 mmol/L, and urine osmolality 110 mosmol/kg. A water deprivation test showed partial diabetes insipidus. The remaining pituitary assessment was hampered by her prednisolone therapy and the oral contraceptive pill. Prolactin and thyroid function were normal. An MRI of the pituitary demonstrated a diffusely enlarged gland containing a poorly enhancing lesion with midline supra-sellar extension, consistent with WG. There was loss of the usual high signal within the posterior gland (Figure 1). She was diagnosed with diabetes insipidus secondary to WG and was initiated on long term desmopressin which controlled the symptoms of diabetes insipidus. A course of intravenous cyclophosphamide and methylprednisolone was administered (monthly pulses of 15 mg/kg and 10 mg/kg, resp., for six months), and oral prednisolone was increased to 60 mg daily. Following this course of treatment a repeat MRI showed a small but definite decrease in the size of the pituitary lesion. 

 Over the next year immunosuppression comprised MMF 1 g twice daily, oral prednisolone (reducing regime), and co-trimoxazole. When her prednisolone dose was reduced below 25 mg daily, however, headaches recurred, and an MRI scan revealed further pituitary enlargement, with a prominent low signal focus in the center, in addition to loss of high signal in the posterior gland. ENT symptoms remained controlled and inflammatory markers low with cANCA of 1 in 40. Rituximab was administered in July 2005 as per case 1 and maintenance immunosuppression reduced to tapering prednisolone. Headaches improved, and her disease was subsequently managed with low-dose prednisolone (10 mg) monotherapy. She has received 2 further treatments with Rituximab, each 12 months apart, for recurrence of symptoms, mainly headache, with good symptomatic improvement each time. Maintenance prednisolone was continued. The pituitary gland showed a reduction in size on MRI after the second course of Rituximab.

## 4. Case 3

In 1995 a 26-year old man presented with malaise, nose bleeds, and sinusitis. cANCA was 1 in 320 (MPO and PR3 not available) and nasal biopsy consistent with WG, but no other organ involvements were identified. He received 9 pulses of IV cyclophosphamide (0.6 mg per m^2^) over 15 months, oral prednisolone, and co-trimoxazole. His disease remained active, and he subsequently developed subglottic/right upper lobe bronchus stenosis. In view of persistently active sinus and upper airways disease, ultimately leading to nasal bridge collapse, he received 6 courses of the humanised monoclonal antilymphocyte antibody alemtuzumab between 1996 and 2002 as well as a short course of oral cyclophosphamide in 1999. His subglottic stenosis required steroid infiltration and dilatation. His disease was subsequently controlled with a small dose of prednisolone (9 mg daily). 

At routine follow-up in 2004 he gave an eight-month history of “socially disabling” polyuria and polydypsia. Neurological examination was normal. Investigations showed ESR 10 mm/h, CRP 19 mg/L, cANCA 1 in 20, serum sodium 140 mmol/L, serum creatinine 98 *μ*mol/L,serum osmolality 301 mosmol/kg, and urine osmolality 139 mosmol/kg (urinary sodium not available). Anterior pituitary testing was within normal limits except TSH 0.05 mIU/L (on thyroxine replacement). 

 MRI showed a diffusely enlarged pituitary and thickened stalk consistent with inflammatory hypophysitis but no enhancing lesion. There was extensive involvement of the sphenoid sinus with inflammatory tissue and a perforated nasal septum. He was seen by an endocrinologist and diagnosed with diabetes insipidus secondary to WG and initiated on long term desmopressin which improved his symptoms of diabetes insipidus. A further course of alemtuzumab was administered. A subsequent MRI was unchanged, but his ENT symptoms improved markedly. Maintenance immunosuppression comprised MMF 1g twice daily and prednisolone 20 mg daily. MMF was discontinued at the patient's request in May 2005. In December 2005 ENT symptoms returned and MRI suggested progressive sinus inflammation and further pituitary enlargement, for which he received a further course of pulsed intravenous cyclophosphamide and methylprednisolone. Subsequently he received two courses of rituximab (each course 2 × 1 g iv, 15 days apart) to control progression of his disease.

## 5. Discussion

Wegener's granulomatosis is a systemic, necrotizing granulomatous vasculitis which can affect virtually any organ in the body. Involvement of the nervous system occurs in up to one third of patients, the commonest neurological presentations being peripheral neuropathies and mononeuritis multiplex. CNS involvement is less frequent but has been documented to cause cranial nerve palsies, cerebral vascular events, and cerebral vasculitis [1]. Wegener's granulomatosis affecting the posterior pituitary is rare with less than 50 cases reported in the international medical literature since 1953, and anterior pituitary involvement is even less common. 

 All three cases described in this report had predominantly ENT disease which remained variably symptomatic, but pituitary involvement developed despite apparent disease control. In each case, posterior pituitary pathology was diagnosed following development of polyuria and polydypsia. On review of literature, the diagnosis of WG typically predates the onset of diabetes insipidus, although hypopituitarism or diabetes insipidus can be a presenting feature [3–8]. The extent of systemic disease varies, and there is no clear pattern which predisposes to pituitary involvement in WG: ENT disease is not present in all cases. 

 Despite responding to therapy, most cases have an ongoing requirement for desmopressin; anterior pituitary involvement is similarly unlikely to recover with treatment. Anterior pituitary function should be monitored in all patients with posterior gland involvement, although assessment may be hampered by corticosteroid therapy. Once anterior pituitary dysfunction is diagnosed, it should be assumed that patients are steroid dependent, and corticosteroid dose should fall no lower than 7.5 mg of prednisolone daily, or equivalent. 

 MRI of the pituitary is useful in diagnosis. The classic MRI findings in WG are diffuse or focal infundibular thickening and the absence of the normal high-intensity signal in the posterior pituitary lobe, seen on T1-weighted images (Figure 1) [9]. These MRI abnormalities may vary with disease activity [3, 5, 7, 10, 11]. Only one of our cases showed classical MRI abnormalities, which improved with initial treatment. All three patients received either oral or pulsed iv cyclophosphamide in conjunction with corticosteroids, a regime which induces remission in up to 75% of WG cases [2]. Twenty case reports of WG and DI were reviewed in which, 12/20 patients received cyclophosphamide with complete resolution of symptoms and signs of vasculitis. 5/20 had alternative regimes (prednisolone alone or with methotrexate), and 3/20 were not published in the English language. Cyclophosphamide has greatly reduced mortality in WG, but there are associated toxicities, not all patients respond, and relapse occurs in some. A number of new or experimental therapies have been advocated in such cases [12]. Our three cases each received a biologic agent for persistent disease or relapse. Rituximab is a chimeric anti-CD20 monoclonal antibody (mAb) which depletes B-lymphocytes but spares plasma cells. It was originally developed for the treatment of B cell lymphomas but has now been used to treat a variety of autoimmune conditions. There are a number of reports of rituximab being used to treat WG with varying success [13–15]. Two of our cases received rituximab with beneficial outcome. Our third case received several courses of alemtuzumab with improvement on each occasion; subsequently he received rituximab for relapsing disease following tapering of immunosuppression. Alemtuzumab is a humanised anti-CD52 mAb which was also initially developed for treatment of haematological malignancy [16]. The CD52 antigen is abundantly expressed on all lymphocytes, and at lower levels on monocytes. Biologic therapies offer a more targeted approach to disease management of systemic vasculitis. Case series reveal a potent effect in refractory disease with an acceptable rate of toxicity, although relapse can occur [17]. The efficacy of this approach alludes to the central role of lymphocytes (B and T cells) in the disease process. Current trials are looking at the earlier introduction of biologics in diseases such as WG. 

 In conclusion, diabetes insipidus resulting from WG is an unusual, but well-described complication and clinicians should have a high index of suspicion when patients present with polyuria and polydypsia. We have presented 3 patients with established WG who subsequently developed diabetes insipidus. All three cases were resistant to traditional treatment but responded, in two cases robustly, to biologic therapies.
